# Adhesive Intestinal Obstruction in Infants and Children: The Place of Conservative Treatment

**DOI:** 10.5402/2011/645104

**Published:** 2011-06-30

**Authors:** Ahmed H. Al-Salem, Mohammad Oquaish

**Affiliations:** Department of Pediatric Surgery, Maternity and Children Hospital, P.O. Box 61015, Dammam, Qatif 31911, Saudi Arabia

## Abstract

*Objectives*. Adhesive intestinal obstruction (AIO) is rare in the pediatric age group and its treatment is still controversial. This is a retrospective review of our experience in infants and children with AIO. *Patients and Methods*. The records of infants and children with AIO between January 2001 and December 2010 were retrospectively reviewed for age at diagnosis, sex, initial operation, interval between initial operation and presentation, diagnosis, treatment and outcome. *Results*. 44 infants and children were admitted with AIO. There were 28 males and 16 females who had 46 episodes. Their ages at presentation ranged from 1 month to 12 years (mean 5.4 years), while their ages at initial operation ranged from 2 days to 12 years (mean 4.15 years). Time elapsed from initial operation to presentation ranged from 7 days to 8 years (mean 1.5 years), and 66% developed AIO within 1 year from initial operation. Appenedecectomy was the commonest operation (29.5%). Four (9%) responded to conservative treatment. The other 40 (91%) required surgical intervention. Twenty-nine had release of adhesions only, while 10 (25%) had resection of small intestines and one underwent stricturoplasty. Two developed recurrence and one died. *Conclusions*. AIO is rare in the pediatric age group and the majority becomes symptomatic within 1 year of operation. Appendecectomy is the commonest operation leading to AIO. The place of conservative treatment is limited and to obviate delay and decrease the chance of intestinal ischemia, they should be treated early with surgical adhesiolysis.

## 1. Introduction

Postoperativeadhesions are almost inevitable after most abdominal surgeries. The severity and extent of adhesions are, however, variable, and, fortunately, the majority of adhesions do not manifest clinically. In spite of this, adhesive intestinal obstruction is common in adults and considered the second cause of intestinal obstruction after obstructed abdominal wall hernias and a major cause of morbidity and significant health care costs [1]. This, however, is not the case in the pediatric age group, where adhesive intestinal obstruction is considered to be rare, and, because of this, the treatment is still controversial [2–5]. The outcomes of conservative treatment are variable, and a balance has to be made between this and surgical adhesiolysis which is also known to be complicated by a high rate of adhesion reformation [3, 4, 6]. This is an analysis of our experience with 44 infants and children with adhesive intestinal obstruction outlining the causes, treatment, and outcome.

## 2. Patients and Methods

The medical records of all infants and children admitted to our hospital between January 2001 and December 2010 with the diagnosis of adhesive intestinal obstruction were retrospectively reviewed. The following information was extracted: age at diagnosis of adhesive intestinal obstruction, sex, age at initial operation, time interval from the initial operation to the development of adhesive intestinal obstruction, type of initial operation, duration of symptoms, type of treatment, and outcome. The diagnosis of adhesive intestinal obstruction was based on a history of previous abdominal surgery, presentation with abdominal pain, vomiting and abdominal distension, failure to pass flatus or stool and the presence of dilated bowel loops with multiple air/fluid levels on supine and erect abdominal radiographs. In all infants and children the treatment was initially conservative and consisted of (1) resuscitation with intravenous fluids and electrolytes, (2) nil by mouth, (3) nasogastric aspiration, and (4) close observation. During observation, the following were recorded: temperature, abdominal girth, abdominal examination for localized tenderness and bowel sounds every 4–6 hours, daily CBC and plain supine and erect abdominal radiographs. The amount and quality of nasogastric aspirate were also recorded. The frequency of these observations was modified according to the response in each case. Conservative treatment was continued for those who showed response in the form of decrease in the amount of nasogastric aspirate, no fever, no leukocytosis, no localized abdominal tenderness, and passage of flatus or feces. The presence of localized abdominal tenderness, fever, and leukocytosis in the absence of any other cause and/or evidence of complete intestinal obstruction that is persisting or free peritoneal air were considered indications for surgery.

## 3. Results

A total of 44 infants and children with the diagnosis of adhesive intestinal obstruction were admitted to our hospital. There were 28 males and 16 females who had 46 episodes of adhesive intestinal obstruction. Their ages at the time of presentation with adhesive intestinal obstruction ranged from 1 month to 12 years (mean 5.4 years and median 5.3 years), while their ages at initial operation ranged from 2 days to 12 years (mean 4.15 years and median 2 years). Time elapsed from initial operation to presentation ranged from 7 days to 8 years (mean 1.5 years and median 4 months), and 66% of our patients developed AIO within 1 year from initial operation. The operative procedures leading to adhesive intestinal obstruction are shown in Table 1. Appendectomy was the commonest operation (29.5%) leading to adhesive intestinal obstruction in our series. This was for perforated acute appendicitis in 5 and acute simple appendicitis in the other 8. This is followed by Hirschsprung's disease following multiple operations. Three of our patients had Wilms' tumor. In one of them this was following a right-sided ruptured Wilms' tumor. Necrotizing enterocolitis and congenital intestinal atresia were responsible for 4 episodes, respectively. In all necrotizing enterocolitis, there was intestinal gangrene with perforation necessitating laparotomy and intestinal resection. Insertion of ventriculoperitoneal shunt was responsible for 3 episodes of adhesive intestinal obstruction. This was for hydrocephalus in 2 and for hydrocephalus secondary to a brain abscess in one. Four (9%) of our patients only responded to conservative treatment. One of them was 15 years old male with sickle cell disease who had splenectomy and cholecystectomy for splenic sequestration crisis and gallstones. Postoperatively he developed hematoma at the splenic bed which required reexploration. Ten days postoperatively, he developed adhesive intestinal obstruction which responded to conservative treatment. The other patient was a 9-year-old girl who was involved in a road traffic accident and had exploration laparotomy in another hospital. She presented 9 years later to our hospital with features of adhesive intestinal obstruction. She responded to conservative treatment. The remaining two patients were 12-year and 11-year-old females who had appendectomy for simple acute appendicitis and developed adhesive intestinal obstruction one year and 1 week postoperatively. The other 40 (91%) children required surgical intervention. In two of them there was a single band causing intestinal obstruction, while the other 38 had multiple adhesions. Twenty-nine of them required release of adhesions only, while 10 (25%) required resection of small intestines and one of them required strictureplasty only. Six had appendectomy and 4 underwent resection of an incidentally found Meckel's diverticulum. Three of our patients had Nobel's placation. Two of our operated patients developed recurrence. One was following laparotomy for necrotizing enterocolitis and presented two months following the initial release of adhesions and resection of small intestines with features of adhesive intestinal obstruction. This also did not respond to conservative treatment and necessitated laparotomy with release of adhesions but no bowel resection. The other patient was 5.5 years old and had appendectomy. He presented 23 days following laparotomy for adhesive intestinal obstruction with readhesions that did not respond to conservative treatment. He underwent laparotomy with release of adhesions and no resection of intestines. One of our patients died giving an overall mortality of 2.3%. A 1-year-old male developed hydrocephalus secondary to a brain abscess and a ventriculoperitoneal shunt. He developed AIO two months after insertion of the shunt. He underwent laparotomy and release of adhesions, and, postoperatively, he did well but died of other nonrelated causes.

## 4. Discussion

Adhesions continue to be a common consequence of abdominal surgery with serious morbidity and occasional cause of death as well as an economic burden [1]. The etiology of adhesion formation remains incompletely understood, and, in spite of advances in surgical techniques, there is little change in the epidemiology of adhesions. This is even so in the era of laparoscopic surgery. Adhesions continue to be mysterious not only in terms of their occurrence but also because of their complications which can occur as early as few days after surgery or remain dormant for several years after the initial procedure. This was the case in our series where we saw adhesive intestinal obstruction as early as 1 week following, surgery and as late as 12 years following the initial operation but, in 66% of our patients, adhesive intestinal obstruction occurred within 1 year from the initial operation. In Janik et al. series, adhesive intestinal obstruction developed within 2 years in 80% of their patients, and in another series adhesive intestinal obstruction developed within 3 months from the initial operation [3, 4]. The reason for this variation is not known. The causes predisposing to adhesive intestinal obstruction are also variable, but appendectomy continues to be the commonest cause. In a series of 871 children who had appendectomy, 1.3% of them developed adhesive intestinal obstruction, and this was the highest (3.4%) in those who had perforated appendicitis [7]. This was also the case in our series. The reason for this is not exactly known, but infection is considered one of the common triggers for adhesion formation. Another contributing factor for adhesion formation is multiple operations as in patients with Hirschsprung's disease who are usually subjected to several operations. This is not related to the original operation but rather to the fact that there was more than one operation. This is specially so if there was infection or contamination. Multiple operations are known to be associated with increased deposition of fibrin which tends to form bridges between adjacent tissues leading to adhesions which can be degraded by the normal fibrinolytic factors. This, however, is not the case always, and surgery, infection, and hypoxia are known to diminish the fibrinolytic activity. The rapid wound healing in children may be the reason for the low incidence of adhesions in children when compared to adults. The exact incidence of adhesive intestinal obstruction in children is not known but has been reported to vary from 2.2% to 8.3% [2, 7]. This is in contrast to adults, where in many counties adhesive intestinal obstruction is considered the second commonest cause of intestinal obstruction after obstructed abdominal wall hernias.

The treatment of adhesive intestinal obstruction is still controversial. Conservative treatment forms the basis for the management of adhesive intestinal obstruction both in children and adults. In the pediatric age group, the response to this is, however, variable. Akgur et al. reported a 40% overall success rate with conservative treatment in a series of 230 episodes of adhesive intestinal obstruction in 181 children [6]. Vijay et al. reported a 48.6% response to conservative treatment in a series of 74 episodes of adhesive intestinal obstruction, and, in their series, children below 1 year of age responded poorly to conservative management [5]. This, however, was associated with an overall resection rate of 16% and a resection rate of 33% in those who underwent operative adhesiolysis. Festen and Janik et al., on the other hand, reported a low success rate with conservative management of adhesive intestinal obstruction in infants and children [3, 4]. This was the case in our series where only 9% responded to conservative treatment. In our series, there was also a 25% resection rate. It is, however, difficult to say for sure that this resection rate could have been reduced by adapting an early surgical intervention. Considering the low success rate with conservative treatment and the fact that delay in operative treatment is also known to affect the outcome adversely by increasing the morbidity, intestinal resections, hospital stay, and cost, we like others advocate early surgical intervention in infants and children with adhesive intestinal obstruction [2–4]. Early surgical intervention saves the child a great deal of pain and discomfort and allows a quick recovery with early discharge from the hospital. Operative adhesiolysis is, however, known to be associated with a high rate of adhesion reformation as well as the risk of inadvertent enterotomy. We encountered two recurrences only in our series. This, however, does not reflect the actual recurrence as it is difficult to say for sure that there were no other recurrences taking in consideration that adhesions may be treated by specialists other than the initial surgeon. The recent advances in minimal invasive surgery with miniaturization of instruments have made it possible for many of the operative procedures to be carried out laparoscopically both in infants and children. This is including laparoscopic adhesiolysis which was shown to be feasible and safe in experienced hands [8]. Not only this but laparoscopy being less invasive and with its widespread use, it is expected to decrease the incidence of adhesive intestinal obstruction. This, however, needs to be substantiated by future studies.

## Figures and Tables

**Table 1:** Operative procedures leading to adhesive intestinal obstruction.

Initial diagnosis and operation	No. patients	%
Appendectomy	13	29.5%
Hirschsprung's disease	4	9.1%
Duodenal and other intestinal atresias	4	9.1%
Necrotizing enterocolitis	4	9.1%
Wilms' tumor	3	6.8%
Ventriculoperitoneal shunt	3	6.8%
Congenital diaphragmatic hernia	2	4.5%
Intussusception	2	4.5%
Abdominal trauma	2	4.5%
Splenectomy and cholecystectomy	1	2.3%
Patent omphalomesenteric duct	1	2.3%
Hyperinsulinism	1	2.3%
Splenectomy for splenic abscess	1	2.3%
Duodenal perforation	1	2.3%
Congenital bands with intestinal obstruction	1	2.3%
Nissen's fundoplication	1	2.3%
